# Supplementary material for: Comparative assessment of nutritional status in unoperated children with Congenital Heart Defects: Insights from a tertiary pediatric cardiac center in India
Source: PLOS Glob Public Health. 2025 Oct 10;5(10):e0005260. doi: 10.1371/journal.pgph.0005260 (PMC12513622; doi:10.1371/journal.pgph.0005260)
Supplement: Attachment — Submitted filename: Barakaale_Revision.docx [file pgph.0005260.s002.docx]

**Comments on Manuscript PGPH-D-25-00105**

Dear Respected Editor,

Thank you for the invitation to review this manuscript. The authors have undertaken important work addressing the nutritional status of children with Congenital Heart Defects (CHD) in India, a significant public health concern. The study provides valuable insights but requires substantial revision.

**Overall**

This study investigates the important issue of malnutrition in a large cohort of Indian children (0-6 years) with unoperated Congenital Heart Defects (CHD), comparing them to community controls. The findings highlight a significantly higher burden of underweight, stunting, and especially wasting in CHD cases. While the data are valuable, several major revisions are required.

**Major Concerns**

1. **Study Design/Control Group**

The claim of a "population-based" comparison is inaccurate. Controls are a community-based convenience sample (health camps/clinics) and may not represent the general population, potentially underestimating the true disparity (as suggested by comparison to NFHS data).

2. **Missing Multivariate Analysis**

The Methods state multivariate analysis was performed to identify independent factors associated with undernutrition, but these crucial results are missing from the manuscript.

3. **Gender Matching Interpretation**

The text claims gender matching (Line 151), citing a Chi-square p-value of 0.0127. This p-value indicates a *statistically significant difference* in gender distribution between cases and controls, contradicting the claim of matching.

**Minor Concerns**

1. **PAH Interpretation**

The comparison of high PAH *prevalence* in this tertiary center cohort (Line 270) with population *incidence* (Line 269) is potentially misleading. Clarify that the high rate reflects referral bias and case severity concentration.

2. **Methodological Clarity**

Briefly justify using RBSK charts for WHZ > 5 years instead of WHO 5-19y standards (Line 131). Ensure clear definitions distinguish the focus on undernutrition vs. broader malnutrition.

3. **Data Presentation**

Improve clarity of comparison labels (x, y, z, etc.) in Table 3. Ensure all abbreviations in Table 4 are clearly defined in the key.

**Recommendation**

Major Revision Required. The manuscript addresses a significant topic with valuable data, but requires substantial revision to address the concerns regarding study design description/limitations, inclusion of multivariate analysis, and correction of statistical interpretations before further consideration for publication.
