# Supplementary material for: Comparative assessment of nutritional status in unoperated children with Congenital Heart Defects: Insights from a tertiary pediatric cardiac center in India
Source: PLOS Glob Public Health. 2025 Oct 10;5(10):e0005260. doi: 10.1371/journal.pgph.0005260 (PMC12513622; doi:10.1371/journal.pgph.0005260)
Supplement: Attachment — Submitted filename: Response to Reviewers.docx [file pgph.0005260.s006.docx]

**Response to Reviewers:**

**Rebuttal Letter to explain queries in review process:**

**Manuscript ID: PGPH-D-25-00105**

**Journal Requirements:**

1. Please provide separate figure files in .tif or .eps format.

**Response: We have provided the figure files in .tif format as per suggestion along with revised manuscript.**

2. In the online submission form, you indicated that “The datasets generated and/or analysed during the current study are not publicly available due to privacy and ethical considerations. This is an institutional data of health records of paediatric patients affected with Congenital Heart Defect and healthy children which included potential identifiers like anthropometric details and age which are essential for presented analysis. Nevertheless, the data will be available from the corresponding author on reasonable request.”

All PLOS journals now require all data underlying the findings described in their manuscript to be freely available to other researchers, either

1. In a public repository,

2. Within the manuscript itself, or

3. Uploaded as supplementary information.

This policy applies to all data except where public deposition would breach compliance with the protocol approved by your research ethics board. If your data cannot be made publicly available for ethical or legal reasons (e.g., public availability would compromise patient privacy), please explain your reasons by return email and your exemption request will be escalated to the editor for approval. Your exemption request will be handled independently and will not hold up the peer review process, but will need to be resolved should your manuscript be accepted for publication. One of the Editorial team will then be in touch if there are any issues.

**Response: We have availed the raw data as supplementary information without participant identifiers (File name uploaded in ‘other’: Supplementary Info-PGPH-D-25-00105)**

**Reviewer 1**

**Major Concerns**

1. **Study Design/Control Group**

The claim of a "population-based" comparison is inaccurate. Controls are a community-based convenience sample (health camps/clinics) and may not represent the general population, potentially underestimating the true disparity (as suggested by comparison to NFHS data).

**Response: Thank you very much for this valuable suggestion. We have corrected the manuscript to refer to a community based convenience sample study at following places:**

| **Line No** | **Section** | **Page No** |
| --- | --- | --- |
| **3-4** | **Title** | **1** |
| **34,40-41** | **Abstract** | **2** |
| **106** | **Deleted in Material and methods** | **4** |
| **118-121** | **Material and methods** | **5** |
| **143** | **Results** | **6** |
| **163-164** | **Results** | **8** |
| **211** | **Discussion** | **11** |
| **329** | **Conclusion** | **15** |

**Thank you for this valuable point.**

**Our analysis considered a significantly large number of health records (n= 11894) from all community based government health centres and primary health care centres in defined geographies. To depict the representativeness of our population controls clearly, we have rectified our statements in manuscript (Page 5, line 118-121). Thus, this is an inclusive sampling method rather than a convenience sampling technique.**

**Thus this community-based data provided the most practical and accessible control group available. In order to address this limitation, we have compared our findings with published large-scale population datasets (NFHS) from Government of India to provide additional context and better estimate the generalizability of our findings.**

2. **Missing Multivariate Analysis**

The Methods state multivariate analysis was performed to identify independent factors associated with undernutrition, but these crucial results are missing from the manuscript.

**Response: Thank you for bringing this oversight to our attention.** **In absence of certain matching independent variables in cases and control datasets, we were unable to perform multivariate analysis. Hence, we have deleted the same from ‘Statistical Analysis’ section.**

3. **Gender Matching Interpretation**

The text claims gender matching (Line 151), citing a Chi-square p-value of 0.0127. This p-value indicates a *statistically significant difference* in gender distribution between cases and controls, contradicting the claim of matching.

**Response: We apologize for this important oversight and sincerely thank the reviewers for bringing it to our attention. We have made this correction in revised file (Result section- Line 150-152, page 6).**

**Minor Concerns**

1. **PAH Interpretation**

The comparison of high PAH *prevalence* in this tertiary center cohort (Line 270) with population *incidence* (Line 269) is potentially misleading. Clarify that the high rate reflects referral bias and case severity concentration.

**Response: Suggestion well taken. The quoted incidence is from a previous report in literature. We have corrected the same in manuscript (Line 272-274).**

2. **Methodological Clarity**

Briefly justify using RBSK charts for WHZ > 5 years instead of WHO 5-19y standards (Line 131). Ensure clear definitions distinguish the focus on undernutrition vs. broader malnutrition.

**Response:**

**Thank you for this critical observation. Indian Government child health initiatives like RBSK (Rashtriya Bal Swasthya Karyakram) use WHO growth charts for reference. Thus RBSK and WHO charts are one and the same. We have made the correction in previous version.**

3. **Data Presentation**

Improve clarity of comparison labels (x, y, z, etc.) in Table 3. Ensure all abbreviations in Table 4 are clearly defined in the key.

**Response:**

**We have deleted x,y,z from table 3 have made appropriate corrections to improve clarity. For table 4, we have rechecked that all abbreviations are mentioned in key.**

**Reviewer 2:**

1. The article meets publication criteria. The abstract succinctly summarizes the study’s objectives, methods, key findings, and implications. However, it could better highlight the novelty of the study (being the first large-scale Indian study comparing CHD patients with population controls).

**Response: Thank you for your positive feedback. We have modified the abstract as per your suggestion.**

2. The introduction effectively establishes the significance of CHD and malnutrition as public health issues, citing global and Indian prevalence data.
The justification for the study is strong, emphasizing the lack of Indian population-based comparisons and the clinical implications of malnutrition in CHD.
The study fills a gap by providing comparative data on malnutrition in CHD vs. healthy children, which is scarce in low-resource settings. The retrospective case-control design is appropriate for comparing nutritional status between CHD patients and controls. Data from a tertiary cardiac center, ensuring clinical validity. Population-based controls from health camps improve generalizability.
Statistical Analysis, Appropriate use of Chi-square, t-tests, and multivariate analysis. However, details on adjustments for confounders (e.g., socioeconomic status) could be clearer.

**Response: Thank you for your kind evaluation of our work. In absence of objective measures of certain matching independent variables in cases and control datasets (e.g. socioeconomic status details were absent in Control dataset), we were unable to perform multivariate analysis. Hence, we have deleted the same from ‘Statistical Analysis’ section.**

3. Limitations- The retrospective design may introduce selection bias, Lack of detailed dietary intake or feeding difficulty data. Controls from health camps may not fully represent the general population.

This study provides valuable insights into malnutrition in Indian CHD patients, with robust methodology and significant public health implications. However, its retrospective nature and lack of granular data on dietary and socioeconomic influences limit deeper mechanistic insights. The findings underscore the need for integrated nutritional interventions in CHD care.

Recommendations
- Prospective longitudinal studies to assess causality.
- Inclusion of dietary and socioeconomic factors.
- Subgroup analysis by CHD type and severity.

**Response: We agree with reviewer completely on this. In discussion, we have accordingly addressed these limitations, strengths of this study and future perspectives in discussion section: line 304-320- page 14.**

**Additionally, we would like to notify that while revisiting our analysis and manuscript, we have reclassified CHD sub groups our data in Table 2 as per clinician’s guidance. Accordingly, the analysis fields have been rectified in Table 5 and results section. Our interpretations and conclusion nevertheless remain the same.**
