# Supplementary material for: Comparative assessment of nutritional status in unoperated children with Congenital Heart Defects: Insights from a tertiary pediatric cardiac center in India
Source: PLOS Glob Public Health. 2025 Oct 10;5(10):e0005260. doi: 10.1371/journal.pgph.0005260 (PMC12513622; doi:10.1371/journal.pgph.0005260)
Supplement: Attachment — Submitted filename: Comments on revised manuscript.docx [file pgph.0005260.s004.docx]

13-7-2025

Dear Editor,

Re: PGPH-D-25-00105R1

Thank you for inviting me to review the revised version of the article titled “Comparative assessment of Nutritional Status in unoperated patients with Congenital Heart Defects: Insights from a Tertiary Paediatric Cardiac Center in India”.

This is a useful and recommended study as it highlights the high burden of some of the various forms of malnutrition, specifically undernutrition, in children in India, the location of the study. It is the first large scale study from India comparing patients in hospital with normal controls in the community.

The subject of malnutrition remains a public health concern globally.

The study will contributes to the knowledge pool by filling the gap as it relates to children with Congenital Heart Defects (CHD), especially the details of their nutritional status.It also draws attention to the need for appropriate intervention in patients with CHD.

*The authors have addressed the comments of the reviewers and I agree with the comments of the reviewers.

I have the following few comments:

Title	Perhaps “Unoperated Patients” can be left out from the title as the work will still fulfil the same purpose.

Abstract: In the abstract, the abbreviation PAH should written in full before its subsequent use.

There was no previous explanation of the abbreviation earlier in the abstract.

And the fact that they were patients assessed preoperatively be stated in the methodology as the authors had already done.

Methodology: In line 109, the word ‘population’ has been deleted for the control group

as one of the reviewers had suggested and which I strongly agree with and the word community can be included..

General: Minor English editing can be done.

**Remark**: It will be of interest to the reading public.
