# Supplementary material for: Comparative assessment of nutritional status in unoperated children with Congenital Heart Defects: Insights from a tertiary pediatric cardiac center in India
Source: PLOS Glob Public Health. 2025 Oct 10;5(10):e0005260. doi: 10.1371/journal.pgph.0005260 (PMC12513622; doi:10.1371/journal.pgph.0005260)
Supplement: Attachment — Submitted filename: Response to Reviewers- 2.docx [file pgph.0005260.s007.docx]

**Response to Reviewers: Revision 2**

**Rebuttal Letter to explain queries in review process:**

**Manuscript ID: PGPH-D-25-00105R1**

**Journal Requirements:**

**1. If the reviewer comments include a recommendation to cite specific previously published works, please review and evaluate these publications to determine whether they are relevant and should be cited. There is no requirement to cite these works unless the editor has indicated otherwise.**

**Response:** Noted with thanks. Since there was no such specific recommendation from reviewers, we have not added any further reference.
 **2. Please review your reference list to ensure that it is complete and correct. If you have cited papers that have been retracted, please include the rationale for doing so in the manuscript text, or remove these references and replace them with relevant current references. Any changes to the reference list should be mentioned in the rebuttal letter that accompanies your revised manuscript. If you need to cite a retracted article, indicate the article’s retracted status in the References list and also include a citation and full reference for the retraction notice.**

**Response:**

a) Suggestion well taken. We didn’t find any retracted article in our reference list.
b) Since article 3 in bibliography was repeated at serial no. 27, we have re-numbered the reference list from 27^th^ onwards and accordingly have made corrections to the manuscript.
c) Since we have also reorganized the discussion section for better understanding and to enhance its logical flow, citations and reference list has been accordingly modified in the prescribed format.

***Kindly refer to clean version of Revision 2 submitted along with this manuscript for mentioned line numbers ahead.**

**Response to Comments on Manuscript( word document in attachment):**

**Dear Editor,**

**Re: PGPH-D-25-00105R1**

**Thank you for inviting me to review the revised version of the article titled “Comparative assessment of**

**Nutritional Status in unoperated patients with Congenital Heart Defects: Insights from a Tertiary**

**Paediatric Cardiac Center in India”.**

**This is a useful and recommended study as it highlights the high burden of some of the various forms of**

**malnutrition, specifically undernutrition, in children in India, the location of the study. It is the first large**

**scale study from India comparing patients in hospital with normal controls in the community.**

**The subject of malnutrition remains a public health concern globally.**

**The study will contributes to the knowledge pool by filling the gap as it relates to children with**

**Congenital Heart Defects (CHD), especially the details of their nutritional status. It also draws attention to the need for appropriate intervention in patients with CHD.**

***The authors have addressed the comments of the reviewers and I agree with the comments of the**

**reviewers.**

**I have the following few comments:**

**1. Title Perhaps “Unoperated Patients” can be left out from the title as the work will still fulfil the same**

**Purpose.**

**Response:** Thank you for the suggestion. We included the term *“Unoperated”* in the title to emphasize that the patients were newly registered in our OPD and had not yet undergone any surgical or catheter-based interventions at our any other centre previously. This distinction was important to ensure that our analysis was based on baseline observations from patients with unrepaired CHD, thereby avoiding potential treatment-related bias. If acceptable to reviewers, we would like to retain the term *‘Unoperated’* in the title to accurately reflect the study population and maintain clarity regarding the absence of treatment-related bias.

We would like to notify instead of *Unoperated ‘Patients’* our title now includes *Unoperated ‘Children’* to be more precise.

**2. Abstract: In the abstract, the abbreviation PAH should be written in full before its subsequent use.**

**There was no previous explanation of the abbreviation earlier in the abstract.**

**And the fact that they were patients assessed preoperatively be stated in the methodology as the authors**

**had already done.**

**Response:** Thank you for this observation. The full form has been added to revised abstract. The patients were assessed preoperatively has been indicated on [page 5, line 113-115 (‘*For cases, unoperated children with CHD were defined as those in the age group 0-6 years having isolated or complex structural cardiac abnormalities present since birth and who had not undergone any surgical procedure or catheter intervention for correction of the same*’), line120 *(‘included reports of pre-operative physical examination and Echocardiography)’*]

**3. Methodology: In line 109, the word ‘population’ has been deleted for the control group**

**as one of the reviewers had suggested and which I strongly agree with and the word community can be**

**included.**

**Response:** Thank you for the suggestion. We have corrected this throughout the manuscript.

**4. General: Minor English editing can be done.**

**Response:** Suggestion well taken. We have worked on it in revision to eliminate possible errors.

**Remark: It will be of interest to the reading public.**

**Reviewer #1:**

**1. Clarity of Gender Matching:**

**There is a discrepancy with regard to gender matching. According to Line 39 of the updated abstract, "age (0-6 years) & gender matched controls." However, it is evident from the data (Table 1, Lines 150–152) that "Gender difference was significant in cases and controls... Cases have 1.22 times higher proportion of males... (p=0.0127)." Lines 311-313 of the discussion further suggest that "gender matching of cases and controls was not feasible."**

**Response:** Thank you for this suggestion. As can be verified in the attached revised manuscript file with this letter, we have deleted *‘& gender matched’* in the revision 1. Hence as stated in main manuscript, gender matching was not feasible as quoted on page 15; line 331-332 of manuscript revision 2 (‘*Our analysis revealed that males affected with CHD were 1.22 times more than female patients. Thus gender matching of cases and controls was not feasible as it would have led to substantial loss of valuable female control information’*).

**2. Statistical Reporting and Data Presentation: The statement "indicating significant undernutrition in cases (p<0.05) compared to age (0-6 years) matched controls" is followed by the chi-square value "(χ2= 6.68, p=0.009)" which is bit unclear. To what particular comparison is this chi-square applied? Is it a test for one specific indicate or for variations in nutritional status overall? The significance for WAZ, HAZ, and WHZ derived by the Z-tests mentioned later for Table 4 may be clearer to understand.**

**Response:**

a) Thank you very much for this critical observation and we agree with reviewer on this. To avoid ambiguity in interpretation, we have revised this statement in abstract and now have mentioned the individual Z values and significance values for WAZ, HAZ, and WHZ derived by Z-test in the abstract.
b) P - values in **Table 4** and in result section of manuscript [page 8, line 171-172 (‘*There was significantly more wasting (Z= 39.01, p<0.01), underweight children (Z= 27.38, p<0.01) and stunting (Z= 8.32, p<0.01) in case group as compared to controls from community in the same age group (0-6 years’)*; page 12, line 258 *(‘cases as compared to controls (14.04%) (p<0.01)’*)] also have been revised to p <0.01 for all three parameters.

**3. Table 4: "Blanks" Give a brief explanation of how "Blanks"—missing data for WAZ, HAZ, or WHZ—were handled in the percentage calculation in the table footnote or methods. For the specific Z-score calculation, were they not included in the denominator?**

**Response:** Thank you for this comment. We have mentioned the explanation in revised manuscript in bracket in key [ page 5, line 133-134 *(‘Incomplete entries or missing values in the data sets were not considered in respective analyses’*), page 10, line 203 *(*‘Not considered in denominator for respective percentage calculations in each category of wasting, stunting and underweight’)* ].

**4. Minor Suggestions:**

**Correct typographical error: "Congenial" to "Congenital" (Line 30).
Line 244 (Discussion): The Kuppuswamy scale citations (21)(22)[(23) – the last (23) is outside the bracket. Correct formatting.**

**Response:** Thank you for your suggestions. We have done these corrections.

**Reviewer #3:**

**The authors raised an important and interesting topic—studying undernutrition in children with congenital heart defects (CHDs) using an observational retrospective case-control design (Cases: Children diagnosed with CHDs. Controls: Children without CHDs).**

**Major issues:**

**1. The issue of reverse causality is significant in such kind of study; it is difficult to determine whether CHDs led to undernutrition or vice versa.**

**Response:** Suggestion well taken. The causality needs be viewed according to periconceptional or postnatal period. We have discussed this point on [page 15, Line 323-327 *(‘Another noteworthy point is that, although a significant association between CHD and undernutrition was observed, establishing causality remains a challenge. While periconceptional nutritional insufficiencies in mother may lead to abnormal cardiac development in utero, postnatally CHD may trigger undernutrition in the course of child growth and development due to feeding challenges and increased metabolic demands’)*] in revision.

**2. The rationale for the investigation is not well justified in the background section.**

**Response:** Thank you for this comment. We have highlighted the rationale in the last paragraph of background [page 4, line 93-100 *(‘From literature we understand that India carries a significant dual burden of CHD and childhood undernutrition, yet their interrelationship remains poorly understood. Globally a few reports have documented poor nutrition status in CHD. But there are very few reports to compare observations in CHD cases with community based controls without CHD. The presented study is amongst the first reports from India to compare the nutritional status and undernutrition severity among CHD children from outpatient department (OPD) of a tertiary paediatric cardiac centre in Maharashtra, India with CHD-free controls from community. This study addresses a critical knowledge gap by providing novel evidence on the relationship between CHD and undernutrition, which may have direct implications for designing early screening strategies and tailored nutritional management in pediatric cardiac care’)].*

**3. The eligibility criteria for cases (children with CHDs), and the methods of case ascertainment and control selection, were not adequately described. There is no explanation of who the cases are or how the authors assessed the children with CHDs (methods section). Furthermore, how controls were assessed is not described—which is very important. Controls should not have chronic conditions or syndromes that affect growth.**

**Response:** Thank you for this important suggestion. We have suitably modified the ‘Study Details’ section under ‘Materials and methods’ on page 4 and 5 (line 105-133) with detailed operational definition (page 5, line 113-115) to identify the cases.

**4. The matching criteria were unclear; specifically, the reason for gender matching was not justified, and the number of controls per case was not described.**

**Response:** We are very thankful for your critical analysis. Since gender matching was not possible for this analysis we have addressed this limitation in manuscript on page 15 (line 331-332)- ‘*Our analysis revealed that males affected with CHD were 1.22 times more than female patients. Thus gender matching of cases and controls was not feasible as it would have led to substantial loss of valuable female control information’.* Also, the correction for number of controls per case has been done on [page 4 line 111 *(‘resulting in approximately 7 controls per case, ensuring sufficient statistical power for comparison of outcomes’)*].

**5. The authors did not clearly mention the exposures/predictors, potential confounders, or effect modifiers in their study, even though they used retrospective data.**

**Response:**

a) We acknowledge this limitation and even have mentioned the same on [page 15, line 316-319 *(‘However we acknowledge that this study design restricted the availability of specific comparable variables which may not be routinely recorded, e.g. Specific exposures and predictors or effect modifiers like maternal health status, dietary habits, socioeconomic status or feeding practices, parental addictions and more; thereby limiting analysis based on potential confounders which might affect the nutritional status and CHD in offspring)*].
b) Data about multifactorial exposures and potential confounders was not available from public health records which are mainly from child health screening. We have also made a recommendation for future studies to overcome this limitation on page 15, line 332-334 *(‘To strengthen the evidence base, we recommend conducting prospective longitudinal studies to better assess causal relationships. Future research should also include detailed dietary assessments and socioeconomic details to account for additional influencing factors like duration of existing CHD’)*].

**6. Despite the retrospective nature of the study, unadjusted estimates (such as odds ratios) and, if applicable, confounder-adjusted estimates (AORs) were not reported. It would be helpful to specify which confounders were adjusted for and why they were included in the final analysis. There is a possibility of confounding, and the authors did not clearly specify how they controlled for confounding variables. Important demographic and clinical variables—such as socio-economic status, feeding practices, and comorbid infections—that may affect nutritional status should have been adjusted.**

**Response:** We reiterate that we acknowledge this limitation. This extensive multifactorial information was lacking in child health screening records and hence was not available for comparative case- control analysis. We have presented the extensive analysis from the available data sets. Also, we have indicated odds ratio in revision at relevant places in manuscript (page 6, line 161; page 8, line 173-175; page 10, line 208-209; page 12, line 259-261) and in abstract (page 2, line 39-41).

**7. There is inconsistent use of the terms “undernutrition” vs. “malnutrition.”**

**Response:** Thank you for bringing this to our notice. We assessed the overnutrition status too and have mentioned the same on page 13 (line 273-274). Also, have clarified the umbrella term ‘malnutrition’ on page 3 (line 81-83). Though our study highlights predominant undernutrition in our CHD patients, malnutrition term was retained only wherever deemed relevant.

**8. The findings may not be generalizable beyond the specific population or setting, especially as the study is not representative (one institution ..Sri Sathya Sai Sanjeevani Centre for Child Heart Care & Training in Pediatric Cardiac Skills)**

**Response:** Suggestion well taken. We have addressed this limitation in revision on [page 15, line 322-323 (‘*Although this investigation benefits from large comparative datasets, one more key limitation is that the case data is derived from a single institution, which may affect generalizability of findings’*)].

**Reviewer #4:**

**This study addresses a significant and under-researched topic, particularly in a resource-limited setting where delayed surgical intervention is common. The focus on "unoperated" patients is a major strength, as it captures a population uniquely vulnerable to the long-term metabolic and hemodynamic consequences of CHD. The topic is of great relevance to pediatric cardiologists, nutritionists, and public health professionals working in similar contexts.**

**However:**

**1. The term "unoperated" needs precise definition. Were these patients awaiting surgery? Were they deemed inoperable? Was this their first presentation to the center?**

**Response:** Thank you for this valuable suggestion. The correction has been made in revision on [page 5, line 113-115 *(‘For cases, unoperated children with CHD were defined as those in the age group 0-6 years having isolated or complex structural cardiac abnormalities present since birth and who had not undergone any surgical procedure or catheter intervention for correction of the same’)*].

**2.The timing of the assessment relative to the diagnosis is crucial. A child diagnosed years ago will have a different nutritional trajectory than one diagnosed recently. This potential confounding factor must be mentioned and discussed.**

**Response:** This point has been addressed in discussion on [page 14-15, line 310-312 *(‘We would like to emphasize that all CHD are present since birth though diagnosed at different time points. Being retrospective study, data was available only for given time point. Thus, the nutritional trajectory for cases could not be assessed’)*]. We have ascertained the nutritional status of the patient at first presentation in our OPD.

**3. Please define the abbreviations (CHD, VSD, ASD, etc.) in a footnote of Table 2 for readers outside the field.**

**Response:** Correction done in Table 2 in revision.
